# Self-Myofascial Release Intervention and Mobile App in Patients With Hemophilic Ankle Arthropathy: Protocol for a Randomized Controlled Trial

**DOI:** 10.2196/15612

**Published:** 2020-07-31

**Authors:** Antonio Javier Meroño-Gallut, Rubén Cuesta-Barriuso, Raúl Pérez-Llanes, Elena Donoso-Úbeda, José-Antonio López-Pina

**Affiliations:** 1 Tú. Bienestar 360º Physiotherapy and Medical Center San Javier-Murcia Spain; 2 Department of Physiotherapy European University of Madrid Madrid Spain; 3 Fishemo CEE Spanish Federation of Hemophilia (FEDHEMO) Madrid Spain; 4 Real Fundación Victoria Eugenia Madrid Spain; 5 Department of Physiotherapy Catholic University San Antonio-UCAM Murcia Spain; 6 Optimus Osteopathy and Physiotherapy Clinic Murcia Spain; 7 Department of Basic Phycology and Methodology University of Murcia Murcia Spain

**Keywords:** Hemophilic arthropathy, ankle, self-myofascial release, joint pain, functionality, randomized clinical trial

## Abstract

**Background:**

Hemophilic ankle arthropathy is manifested by degenerative functional alterations and chronic pain. Myofascial release techniques are used to treat soft tissue adhesions, relieve pain, and reduce tissue sensitivity.

**Objective:**

This study aims to evaluate the safety and efficacy of a protocol using self-myofascial release with a foam roller to be applied in patients with hemophilic ankle arthropathy.

**Methods:**

Patients with ankle arthropathy (N=70) will be recruited, enrolled, and assigned to one of two groups—experimental or control—in a 1:1 allocation ratio. Patients will be recruited from 5 centers in different regions of Spain. Patient data will be collected at baseline, posttreatment, and follow-up. The primary outcome will be frequency of ankle joint bleeding (self-reported). The secondary outcomes will be ankle range of motion (measured with a digital goniometer); joint pain (measured with a visual analog scale and an algometer); joint status (measured using the Hemophilia Joint Health Score); muscle strength (measured with a dynamometer); functionality of lower limbs (measured using the 6-minute walking test); activity (self-reported); and muscle flexibility (measured using the fingertip-to-floor test). The treatment program includes 11 exercises that must be administered bilaterally. A mobile app will be developed where each patient will be able to observe the exercises to be carried out. Each session will last 15 minutes with 5 physiotherapy sessions per week for a period of 3 months. It is expected that patients with hemophilia who receive the foam roller intervention will show improvement in mobility, pain, and status of the ankle joint; muscle strength; and function in the lower extremities.

**Results:**

The study has been approved by the institutional review board of the University of Murcia. Patient recruitment will begin in September 2020, and the intervention period will last until June 2021. Data collection will take place between September 2020 and October 2021.

**Conclusions:**

This protocol describes a randomized clinical trial to examine the safety and efficacy of a self-myofascial release intervention using a foam roller in patients with hemophilic ankle arthropathy.

**Trial Registration:**

ClinicalTrials.gov NCT03914287; http://clinicaltrials.gov/ct2/show/NCT03914287.

**International Registered Report Identifier (IRRID):**

PRR1-10.2196/15612

## Introduction

Hemophilia is a congenital coagulopathy characterized by a deficiency of a clotting factor (factor VIII in hemophilia A and factor IX in hemophilia B). Most clinical manifestations are musculoskeletal such as bruising and hemarthrosis. The recurrence of joint bleeding causes degenerative, progressive, and chronic joint deterioration (hemophiliac arthropathy); chronic pain; and a deteriorated perception of the patient's quality of life. Symptoms of hemophilic arthropathy include limited range of motion, decreased periarticular muscle strength, decreased proprioception, and chronic pain [1,2].

The ankle joint, a crucial component of locomotion, is one of the most affected joints (as are the knee and elbow joints) [3], imposing important functional and proprioceptive limitations. These limitations are the result of developing intra-articular alterations such as joint space narrowing, the development of osteophytes, bone deformities, or axial alterations [4]. Prophylactic treatment has been shown to be highly effective in the prevention of hemophilic arthropathy [5]; however, many patients, nevertheless, develop these sequelae as a result of late initiation of treatment, poor adherence to prophylaxis, or the development of inhibitors (antibodies to factor VIII or factor IX concentrates).

The treatment of hemophilic ankle arthropathy includes pharmacologic and orthopedic interventions with the aim of improving symptoms in the patient. The administration of anti-inflammatory and analgesic drugs [6], conducting synoviorthesis to remove the synovial membrane, and ultimately, arthrodesis of the ankle joint are the most common treatments [7].

Physiotherapy can play an extremely important role in the treatment of hemophilic ankle arthropathy. Manual therapy techniques [8], therapeutic exercise [9], or electrotherapy [10] have provided positive results in the control of chronic pain in patients with hemophilic ankle arthropathy.

The degenerative process, itself, is one of the intrinsic mechanisms affecting the fascial system, since it favors loss of elasticity and flexibility, and triggers a process of pathological cross-linking of collagen fibers. Fascial therapy aims to remove fascial tissue restriction through mechanical stimuli applied for 3 to 5 minutes in order to promote the piezoelectric effect in the crystalline matrix of the fascia [11]. The mechanical stimulus to the fascia (in the form of either pressure or tangential compression) triggers a secondary conjunctive cellular response and promotes cellular communication through piezoelectric and mechanotransduction mechanisms [12]. Although pilot and cohort studies [13,14] have proven that this technique is safe in patients with hemophilic arthropathy of the ankle, clinical studies need to be conducted with a large sample size in order to confirm that this technique is indeed suitable.

Fascia is the soft tissue component of the connective tissue matrix that extends through the human body forming a continuous three-dimensional composite of structural support throughout the body [15]. Myofascial release is a physiotherapy technique in which sustained pressure is applied in order to reduce restrictions of the fascia. Mechanical stimulation favors the readjustment of collagen fibers and improves the quality of movement, fluid circulation, and lymphatic drainage [16]. These changes can help break apart adhesions caused by processes such as scarring and fibrosis in the body. Myofascial induction is a manual physiotherapy technique that applies the principles of biomechanical loading of soft tissue and modification of neural reflexes through stimulation of the mechanoreceptors in the fascia to release fascial restrictions and restore healthy tissue [17].

Self-myofascial release with a foam roller is used to treat fascial adhesions and to restore the normal extensibility of soft tissues [18]. Self-myofascial release has similar effects to those provided by manual fascial release techniques. These benefits include easing muscle tension and stiffness, reducing pain, reducing inflammation, alleviating muscle spasms, and increasing joint mobility. Myofascial release techniques have been used to treat adhesions of the soft tissue, to relieve pain and tissue sensitivity, and to reduce edema and inflammation, while improving muscle recovery [19]. In self-myofascial release, patients use their own body weight and materials such as a foam roller to exert pressure on the affected soft tissues [20,21]. Scientific evidence has shown that these types of tools help improve the range of motion as well as pre and postexercise muscle performance [21,22]. The efficacy of these types of exercises to improve joint mobility has been tested in relation to improving mobility in knee flexion [20] and relative to improving ankle joint mobility [23].

The use of foam rollers for myofascial release is based on findings of postintervention changes to joint range of motion and pressure pain thresholds which may be due to a mechanical and neurophysiological responses [24,25]. The direct pressure of the roller may produce local mechanical and global neurophysiological effects that relax the tissues and attenuate pain in the targeted and surrounding area [26]. Local pressure from the roller may affect the viscoelastic properties of the myofascia which may be responsible for such changes within the tissue. Other mechanisms that may be involved include thixotropy (reduced viscosity when stress is applied), reduced myofascial restriction (from the breaking apart of physical adhesions), fluid-related changes (forced fluid displacement within the tissue), and cellular responses (elicited through mechanotransduction from the applied stress within the tissues) [24]. Researchers have also found that foam-rolling reduces arterial stiffness, increases arterial tissue perfusion, and improves vascular endothelial functions related to tissue relaxation [27].

The objective of this paper is to describe our approach to the design and implementation of a protocol for self-myofascial release using a foam roller for patients with hemophilic ankle arthropathy with the aim of reducing the frequency of joint bleeding; improving range of motion; reducing joint pain; and restoring functionality, structural integrity, muscle flexibility, and muscle strength.

## Methods

### Study Design

This study is designed as a randomized controlled multicenter clinical trial (ClinicalTrials.gov; NCT03914287) to establish the safety and efficacy of a physiotherapy intervention consisting of self-myofascial release using a foam roller. Once eligibility is determined, participants will be randomized to either the intervention (experimental) group or the control group.

### Clinical Record and Selection Criteria

This study has been approved by the research ethics committee of the University of Murcia (2428/2019). Patients will be included if they have been diagnosed with hemophilia A or B, are 18 years of age or older, have been diagnosed with bilateral hemophilic arthropathy of the ankle (more than 3 points of joint damage on the Hemophilia Joint Health Score), and are on prophylactic or on-demand treatment with factor VIII or factor IX concentrates.

Patients will be excluded from the study if they are not able to ambulate; have been found to have inhibitors (antibodies to factor VIII or factor IX), have neurological or cognitive alterations that prevent them from understanding the questionnaires and physical tests, or choose not to give informed consent (have not signed the statement of informed consent).

### Sample Size

The target sample size for inclusion in the study is 70 patients. The sample size is justified with respect to the prevalence of patients with congenital coagulopathies; there are 2039 patients with either hemophilia A and B or von Willebrand disease in Spain, of which 612 meet the selection criteria for the study (Spanish Federation of Hemophilia). Based on these data, the sample size at national level would be 236 individuals, with a confidence level of 95% and an expected dropout ratio of 15%. Thus, a sample size of a total of 70 patients from the 5 centers will be used.

### Randomization

Participants will be randomly assignment using opaque envelopes to one of two groups—experimental and control. Random assignment of patients will be carried out after cluster recruitment (based on hemophilia type and patient age) to ensure homogeneity of the study groups. This assignment will be carried out by a person who is not involved in the study objectives and who is unaware of the identity of the participants.

### Outcome Measures

Baseline assessments will be carried out at Hemophilia Association centers. Measurements will be performed by a physiotherapist who is unaware of patient group assignments.

After the treatment period and 3 months after completion, the patients included in both groups (experimental and control) will be re-evaluated by the same rater under the same conditions as their initial evaluation.

Dependent variables (range of motion, joint pain, functionality, joint state, muscle strength, muscle flexibility, and record of activity) will be measured at each study assessment (baseline, posttreatment, and follow-up). A 10-point visual analog scale will be used to evaluate patient perception of joint pain, ranging from 0 (no pain) to 10 (maximum perceived pain). An algometer (FPN100; Wagner Instruments) will be used for the assessment of pressure-induced pain (the amount of pressure at which the person perceives pain), both at the joint and at other body sites [28]. Pressure will be applied gradually at the site, increasing at rate of 50 kPa/s until the patient reports that the sensation is painful [29]. In patients with ankle arthropathy, bilateral measurements will be taken anterior to the lateral malleolus (ankle) [30] and at two other sites—the spinous process of the L5 vertebra and on the extensor carpi radialis longus muscle of the forearm (5 cm distal to the lateral epicondyle of the humerus) [28]. Using a portable instrument [31] for assessing physical activity, patient physical activity level (average number of steps per day, average distance per day, average amount of active time, and energy consumption) will be recorded. The Hemophilia Joint Health Score [32] will be used to evaluate the joint condition of knees, ankles, and elbows. It includes 8 items (inflammation and its duration, pain, atrophy and muscle strength, crepitus, and reduced flexion and extension) ranging from 0 to 20 points per joint (the higher the score, the greater the joint deterioration).

The 6-minute walking test [33] measures the distance walked over a period of 6 minutes as an assessment of submaximal capacity to perform exercise. This instrument was developed for use in patients with respiratory disease and heart failure, but has been used in children and adults with a variety of chronic conditions, including hemophilia [34-36]. The test is performed on a 30 to 50-meter track, and the use of walking aids or orthopedic devices is permitted. Ankle range of motion will be measured by a digital goniometer using a protocol designed by Thornton et al [37]; the assessment can be performed with the patient standing. The axis of the goniometer will be placed alongside the lateral malleolus of the ankle. The fixed arm will be placed parallel to the fibula while the mobile arm is aligned to be parallel with the fifth metatarsal bone. This measuring instrument allows more accurate measurements than those obtained with a plastic goniometer. The patient will be asked to perform 3 repetitions of each movement. The mean of the 3 measurements will be used [28]. The maximum isometric strength of the plantar flexor muscles of the ankle will be evaluated on both limbs with a manual dynamometer. The patient will be placed in a supine position with 90º ankle dorsiflexion. The dynamometer will be located proximal to the metatarsophalangeal joints on the plantar side) and will be held by the evaluator [38]. The patient will be asked to perform 3 maximal effort isometric contractions (each for 5 seconds with a 30-second break in between) against the dynamometer and the mean of the 3 measurements will be used [28]. The fingertip-to-floor test [39] assesses the degree of flexibility of the posterior muscles of the lower limbs. The distance between the fingertips and the floor is calculated at maximum hip flexion with knees extended.

### Intervention

Patients will continue their factor VIII or factor IX concentrate treatment as prescribed by their referring hematologists. Throughout the study, the prescribed pharmacologic treatment, dosage, and periods of substitute treatment of the participants will not be altered. Participants in the control group will receive no physical therapy using self-myofascial release and will continue with their usual routine of physical activity and exercise. Outcomes will be assessed under the same conditions as those of the experimental group.

At the beginning of the study, the main investigator will explain the characteristics of the intervention to be carried out to the experimental group. Each session will last approximately 15 minutes, with 5 physiotherapy sessions over a period of 3 months. Each patient will perform the interventions at home. Participants will have free access to the mobile app. Access to the platform will be approved after the patient signs the informed consent form. Once the study is complete, the contents of the app (Table 1) will be available on the website of the Hemophilia Physiotherapy research group (InHeFis).

The protocol for self-myofascial release of the lower limbs using a foam roller and solid ball massage, adapted to patients with hemophilic ankle arthropathy, will include self-myofascial release of the plantar, back of the leg, and hamstring regions, as well as adductor, abductor, and pelvitrochanteric muscles. Table 1 shows the physiotherapy protocol. Periodic follow-up will be performed, through phone calls and by SMS text messages, to answer questions from patients regarding the use of the app.

A mobile app (designed by the research group) will be used to demonstrate all the exercises in the self-myofascial release protocol. Each time the patient exercises, he or she will be able to watch a video explaining the characteristics of each exercise (time, repetitions, position, and action). This free app will also collect adherence data recorded as days of exercise by the participant.

Patient will need to have a suitable floor mat. A massage ball will be used for pressure point massage (solid ball massage), typically 40 mm to 50 mm in diameter, made of a hard polyurethane, but slightly padded. Two smooth low-deformability foam rollers (90 cm in length and 15 cm in diameter) will also be used.

**Table 1:** Physiotherapy protocol using self-myofascial release for patients with hemophilic ankle arthropathy.

Region and exercise aid		Action	Observations	Times
**Plantar region**				
	Solid ball massage	Slow circular movements shall be carried out with slight pressure on the sole of the foot. The stimulus should be applied below the pain threshold.	Three areas shall be worked on: near the heel, in the middle of the sole of the foot and below the metatarsal head. Circular movements shall be carried out.	5 seconds in each area, bilaterally
	Solid ball massage	Linear movements shall be carried out on the sole of the foot exerting a slight pressure from the heel to the metatarsals and from the metatarsals to the heel.	The maneuver shall include slight flexion and extension movements of the toes.	15 repetitions shall be performed for each foot
**Achilles region**				
	Foam roller	The movement shall be performed from the most distal part of the leg to the region of myotendinous connection with the calf muscles.	The exercise is to be assisted with slight plantar flexion and extension movements by the patient. The exercise can be enhanced by crossing a leg in extension over the leg subject to the work stimulus	15 slow sliding movements
**Soleus and calf muscles**				
	Foam roller	Movement assisted with slight plantar flexion and extension movements shall be performed. The roller will move over the calf muscle.	The exercise can be enhanced by crossing a leg in extension over the leg subject to the work stimulus.	15 slow sliding movements
**Extensor retinaculum of the ankle**				
	Foam roller	With the foam roller placed in the anterior region of the ankle, a sliding motion while exerting slight pressure on the anterior region of the ankle shall be performed.	Performed with the patient in a quadruped position. Slight plantar and dorsal flexion movements of the ankle can be combined.	15 slow movements
**Muscle compartment of the anteroexternal region of the leg**				
	Foam roller	Movements shall be performed on the anteroexternal region of the leg, with slight compression stimuli and longitudinal release of the tibialis anterior, toe extensor, and peroneal muscles.	Performed with the patient in a quadruped position incorporating a slight hip rotation	15 slow movements
**Hamstring region**				
	Foam roller	Bilateral work on the hamstring region using a foam roller shall be performed by rolling each hamstring on the foam roller bilaterally and attempting the widest possible run.	Performed with the patient seated in such a way that the hamstring muscles are supported on the foam roller.	10 slow movements
	Foam roller	Unilateral work on the hamstring region using a foam roller shall be performed focusing the work on one leg while making slight flexion and extension movements of the knee.	Performed with the patient seated in such a way that the hamstring muscles are supported on the foam roller.	10 slow movements
**Adductor muscles**				
	Solid ball massage	The exercise shall be carried out in 3 different areas of the inner thigh region: middle third, proximal third and distal third, starting on the distal third. The ball will be placed between the legs and slight pressure will be applied.	With sustained pressure, slow movements shall be performed with one leg so the solid ball moves in a circle. Movements shall be performed using each leg, changing the position of the ball to the medial third, and then the proximal third.	5 slow movements
**Abductor muscles**				
	Foam roller	The patient shall be positioned in lateral decubitus, with the foam roller on the side of the thigh, applying slow slides on the back of the thigh	The longitudinal run should be as wide as possible.	15 slow movements
**Pelvitrochanteric muscles**				
	Foam roller	The patient shall be seated on the foam roller, flexing a hip while placing the foot with the sole resting on the floor to ensure greater body weight on the gluteal and pelvitrochanteric region on the opposite side.	Perform smooth movements sliding the gluteal region over the foam roller. Then change legs.	15 slow movements

### Data Analysis

The distribution of the sample, the changes after the intervention and follow-up period in each group, and the intra and interindividual effect will be analyzed. We will use the intent-to-treat method to include all individuals who were randomized in the final data analysis.

The statistical analysis will be carried out using SPSS statistical software (version 19.0; IBM Corp). The Kolmogorov-Smirnov test will be used to test for normality, and the Levene test will be used to test for homoscedasticity. If the conditions of normality and homoscedasticity are violated, nonparametric tests will be used.

Descriptive statistics (mean and standard deviation) will be calculated for all dependent variables. A one-way repeated measures analysis of variance will be used to compare the groups (experimental and control) at 3 assessment times (baseline, posttreatment, and follow-up). If the interaction is found to be significant, pairwise comparisons will be performed. The significance level will be set at α=.05. To control the error rate, Bonferroni correction will be applied. The results of the *F* test will depend on the significance of Mauchly sphericity. If significant, the Greenhouse-Geisser correction will be used. To assess clinical relevance, we will calculate the standard error of measurement and minimum detectable change for each dependent variable.

## Results

The study has been approved by the ethics committee of the University of Murcia (ID: 2428/2019). Patient recruitment will begin in September 2020, and an intervention period will continue until June 2021. Data collection will take place between September 2020 and October 2021.

## Discussion

This project is the most ambitious physiotherapy study, in terms of methodology, recorded to date in Spain. The inclusion of 70 patients with hemophilia from 5 different regions for participation in a physiotherapy program will confer on this project a high statistical power, which is unusual in a rare pathology such as hemophilia. Patients with hemophilic ankle arthropathy will undergo treatment with self-myofascial release using a foam roller. The research team is made up of multidisciplinary hemophilia specialists, renowned physiotherapists with extensive clinical experience, and researchers in the field of hemophilia, as well as experts in methodology and statistics. Coordination between different regions of Spain, with the participation of universities, hospitals and associations of patients, is an uncommon effort in the field of hemophilia-related physiotherapy research.

Another potential strength is that this protocol can help to establish a rapid, safe, and effective intervention for patients with hemophilia. In addition to clinical improvements, it could facilitate greater adherence to physiotherapy treatments and improve the quality of life of individuals with hemophilia.

Self-myofascial release requires no economic investment in the case of manual therapy. Validating its safety and efficacy could promote the development of a quick, inexpensive, and simple physiotherapy intervention that can easily be used widely.

## Appendix

Multimedia Appendix 1 CONSORT-EHEALTH checklist (V. 1. 6. 1).
