# Supplementary material for: Self-Myofascial Release Intervention and Mobile App in Patients With Hemophilic Ankle Arthropathy: Protocol for a Randomized Controlled Trial
Source: JMIR Res Protoc. 2020 Jul 31;9(7):e15612. doi: 10.2196/15612 (PMC7428933; doi:10.2196/15612)
Supplement: Multimedia Appendix 1 [file resprot_v9i7e15612_app1.pdf]

CONSORT-EHEALTH (V 1.6.1) - Submission/Publication Form

The CONSORT-EHEALTH checklist is intended for authors of randomized trials evaluating web-based and Internet-based applications/interventions, including mobile interventions, electronic games (incl multiplayer games), social media, certain telehealth applications, and other interactive and/or networked electronic applications. Some of the items (e.g. all subitems under item 5 - description of the intervention) may also be applicable for other study designs.

The goal of the CONSORT EHEALTH checklist and guideline is to be
a) a guide for reporting for authors of RCTs,
b) to form a basis for appraisal of an ehealth trial (in terms of validity)

CONSORT-EHEALTH items/subitems are MANDATORY reporting items for studies published in the Journal of Medical Internet Research and other journals / scientific societies endorsing the checklist.

Items numbered 1., 2., 3., 4a., 4b etc are original CONSORT or CONSORT-NPT (non-pharmacologic treatment) items.
Items with Roman numerals (i., ii, iii, iv etc.) are CONSORT-EHEALTH extensions/clarifications.

As the CONSORT-EHEALTH checklist is still considered in a formative stage, we would ask that you also RATE ON A SCALE OF 1-5 how important/useful you feel each item is FOR THE PURPOSE OF THE CHECKLIST and reporting guideline (optional).

Mandatory reporting items are marked with a red *.
In the textboxes, either copy & paste the relevant sections from your manuscript into this form - please include any quotes from your manuscript in QUOTATION MARKS,
or answer directly by providing additional information not in the manuscript, or elaborating on why the item was not relevant for this study.

YOUR ANSWERS WILL BE PUBLISHED AS A SUPPLEMENTARY FILE TO YOUR PUBLICATION IN JMIR AND ARE CONSIDERED PART OF YOUR PUBLICATION (IF ACCEPTED).
Please fill in these questions diligently. Information will not be copyedited, so please use proper spelling and grammar, use correct capitalization, and avoid abbreviations.

DO NOT FORGET TO SAVE AS PDF _AND_ CLICK THE SUBMIT BUTTON SO YOUR ANSWERS ARE IN OUR DATABASE !!!

Citation Suggestion (if you append the pdf as Appendix we suggest to cite this paper in the caption):
Eysenbach G, CONSORT-EHEALTH Group
CONSORT-EHEALTH: Improving and Standardizing Evaluation Reports of Web-based and Mobile Health Interventions
J Med Internet Res 2011;13(4):e126
URL: http://www.jmir.org/2011/4/e126/
doi: 10.2196/jmir.1923
PMID: 22209829

*Obligatorio

Your First Last

name *

Rubén Cuesta-Barriuso

Primary Affiliation (short), City, Country *

University of Toronto, Toronto, Canada

European University of Madrid, Madrid, Spain

Your e-mail address *

abc@gmail.com

ruben.cuestab@gmail.com

Title of your manuscript *

Provide the (draft) title of your manuscript.

Self-myofascial release using a Foam Roller in patients with hemophilic ankle arthropathy. Protocol for a Randomized Controlled Trial.

Name of your App/Software/Intervention *

If there is a short and a long/alternate name, write the short name first and add the long name in brackets.

Self-myofascial release using Foam Roller

Evaluated Version (if any)

e.g. "V1", "Release 2017-03-01", "Version 2.0.27913"

v1

Language(s) *

What language is the intervention/app in? If multiple languages are available, separate by comma (e.g. "English, French")

Spanish

URL of your Intervention Website or App

e.g. a direct link to the mobile app on app in appstore (itunes, Google Play), or URL of the website. If the intervention is a DVD or hardware, you can also link to an Amazon page.

URL of an image/screenshot (optional)

Accessibility *

Can an enduser access the intervention presently?

- access is free and open
- access only for special usergroups, not open
- access is open to everyone, but requires payment/subscription/in-app purchases
- app/intervention no longer accessible
- Otro:

Primary Medical Indication/Disease/Condition *

e.g. "Stress", "Diabetes", or define the target group in brackets after the condition, e.g. "Autism (Parents of children with)", "Alzheimers (Informal Caregivers of)"

Hemophilia

Primary Outcomes measured in trial *

comma-separated list of primary outcomes reported in the trial

Frequency of ankle joint bleeding

Secondary/other

Are there any other outcomes the intervention is expected to affect?

outcomes

Ankle
range of motion, Joint pain, Joint status, Muscle strength, Functionality of lower limbs, Activity record, Muscle flexibility

Recommended "Dose" *

What do the instructions for users say on how often the app should be used?

- Approximately Daily
- Approximately Weekly
- Approximately Monthly
- Approximately Yearly
- "as needed"
- Otro:

Approx. Percentage of Users (starters) still using the app as recommended after 3 months *

- unknown / not evaluated
- 0-10%
- 11-20%
- 21-30%
- 31-40%
- 41-50%
- 51-60%
- 61-70%
- 71%-80%
- 81-90%

91-100%

Otro:

Overall, was the app/intervention effective? *

yes: all primary outcomes were significantly better in intervention group vs control

partly: SOME primary outcomes were significantly better in intervention group vs control

no statistically significant difference between control and intervention

potentially harmful: control was significantly better than intervention in one or more outcomes

inconclusive: more research is needed

Otro:

Article Preparation Status/Stage *

At which stage in your article preparation are you currently (at the time you fill in this form)

not submitted yet - in early draft status

not submitted yet - in late draft status, just before submission

submitted to a journal but not reviewed yet

submitted to a journal and after receiving initial reviewer comments

submitted to a journal and accepted, but not published yet

published

Otro:

Journal *

If you already know where you will submit this paper (or if it is already submitted), please provide the journal name (if it is not JMIR, provide the journal name under "other")

not submitted yet / unclear where I will submit this

- Journal of Medical Internet Research (JMIR)
- JMIR mHealth and UHealth
- JMIR Serious Games
- JMIR Mental Health
- JMIR Public Health
- JMIR Formative Research
- Other JMIR sister journal
- Otro:

Is this a full powered effectiveness trial or a pilot/feasibility trial? *

- Pilot/feasibility
- Fully powered

Manuscript tracking number *

If this is a JMIR submission, please provide the manuscript tracking number under "other" (The ms tracking number can be found in the submission acknowledgement email, or when you login as author in JMIR. If the paper is already published in JMIR, then the ms tracking number is the four-digit number at the end of the DOI, to be found at the bottom of each published article in JMIR)

- no ms number (yet) / not (yet) submitted to / published in JMIR
- Otro:

TITLE AND ABSTRACT

1a) TITLE: Identification as a randomized trial in the title

1a) Does your paper address CONSORT item 1a? *

I.e does the title contain the phrase "Randomized Controlled Trial"? (if not, explain the reason under "other")

- yes

Otro:

1a-i) Identify the mode of delivery in the title

Identify the mode of delivery. Preferably use “web-based” and/or “mobile” and/or “electronic game” in the title. Avoid ambiguous terms like “online”, “virtual”, “interactive”. Use “Internet-based” only if Intervention includes non-web-based Internet components (e.g. email), use “computer-based” or “electronic” only if offline products are used. Use “virtual” only in the context of “virtual reality” (3-D worlds). Use “online” only in the context of “online support groups”. Complement or substitute product names with broader terms for the class of products (such as “mobile” or “smart phone” instead of “iphone”), especially if the application runs on different platforms.

subitem not at all important 1 2 3 4 5 essential

Does your paper address subitem 1a-i? *

Copy and paste relevant sections from manuscript title (include quotes in quotation marks "like this" to indicate direct quotes from your manuscript), or elaborate on this item by providing additional information not in the ms, or briefly explain why the item is not applicable/relevant for your study

Self-myofascial release with Foam Roller by mobile app in patients with
hemophilic ankle arthropathy. Protocol for a Randomized Controlled
Trial.

1a-ii) Non-web-based components or important co-interventions in title

Mention non-web-based components or important co-interventions in title, if any (e.g., “with telephone support”).

subitem not at all important 1 2 3 4 5 essential

Does your paper address subitem 1a-ii?

Copy and paste relevant sections from manuscript title (include quotes in quotation marks "like this" to indicate direct quotes from your manuscript), or elaborate on this item by providing additional information not in the ms, or briefly explain why the item is not applicable/relevant for your study

1a-iii) Primary condition or target group in the title

Mention primary condition or target group in the title, if any (e.g., “for children with Type I Diabetes”) Example: A Web-based and Mobile Intervention with Telephone Support for Children with Type I Diabetes: Randomized Controlled Trial

subitem not at all 1 2 3 4 5 essential

important

Does your paper address subitem 1a-iii? *

Copy and paste relevant sections from manuscript title (include quotes in quotation marks "like this" to indicate direct quotes from your manuscript), or elaborate on this item by providing additional information not in the ms, or briefly explain why the item is not applicable/relevant for your study

1b) ABSTRACT: Structured summary of trial design, methods, results, and conclusions

NPT extension: Description of experimental treatment, comparator, care providers, centers, and blinding status.

1b-i) Key features/functionalities/components of the intervention and comparator in the METHODS section of the ABSTRACT

Mention key features/functionalities/components of the intervention and comparator in the abstract. If possible, also mention theories and principles used for designing the site. Keep in mind the needs of systematic reviewers and indexers by including important synonyms. (Note: Only report in the abstract what the main paper is reporting. If this information is missing from the main body of text, consider adding it)

subitem not at all important 1 2 3 4 5 essential

Does your paper address subitem 1b-i? *

Copy and paste relevant sections from the manuscript abstract (include quotes in quotation marks "like this" to indicate direct quotes from your manuscript), or elaborate on this item by providing additional information not in the ms, or briefly explain why the item is not applicable/relevant for your study

Methods:
From September 2020 to June 2021, 70 patients with
ankle arthropathy
will be recruited to randomized clinical trial. Participants
will be
assigning to one of two groups: experimental group (n =
35) and control
group (n = 35). Patients will be recruited in 5 centers, from
different
regions of Spain. Patient´s will be collected at baseline,
posttreatment
and follow-up period. Primary outcome will be frequency
of ankle joint
bleeding (self-reported). Secondary outcomes will be ankle
range of
motion (digital goniometer); joint pain (visual analog scale
and
algometer); joint status (Haemophilia Joint Health Score);
muscle
strength (dynamometer); functionality of lower limbs
(6-Minute Walking
test); activity record; and muscle flexibility (Finger-floor
test). The
treatment program includes 11 exercises that must be
administered
bilaterally. A mobile application will be developed where
each patient
will be able to observe the exercises to be carried out.
Each session
will last 15 minutes, with five physiotherapy sessions per
week for a
period of 3 months.

1b-ii) Level of human involvement in the METHODS section of the ABSTRACT

Clarify the level of human involvement in the abstract, e.g., use phrases like “fully automated” vs. “therapist/nurse/care provider/physician-assisted” (mention number and expertise of providers involved, if any). (Note: Only report in the abstract what the main paper is reporting. If this information is missing from the main body of text, consider adding it)

subitem not at all important 1 2 3 4 5 essential

Does your paper address subitem 1b-ii?

Copy and paste relevant sections from the manuscript abstract (include quotes in quotation marks "like this" to indicate direct quotes from your manuscript), or elaborate on this item by providing additional information not in the ms, or briefly explain why the item is not applicable/relevant for your study

1b-iii) Open vs. closed, web-based (self-assessment) vs. face-to-face assessments in the METHODS section of the ABSTRACT

Mention how participants were recruited (online vs. offline), e.g., from an open access website or from a clinic or a closed online user group (closed usergroup trial), and clarify if this was a purely web-based trial, or there were face-to-face components (as part of the intervention or for assessment). Clearly say if outcomes were self-assessed through questionnaires (as common in web-based trials). Note: In traditional offline trials, an open trial (open-label trial) is a type of clinical trial in which both the researchers and participants know which

treatment is being administered. To avoid confusion, use “blinded” or “unblinded” to indicated the level of blinding instead of “open”, as “open” in web-based trials usually refers to “open access” (i.e. participants can self-enrol). (Note: Only report in the abstract what the main paper is reporting. If this information is missing from the main body of text, consider adding it)

subitem not at all important 1 2 3 4 5 essential

Does your paper address subitem 1b-iii?

Copy and paste relevant sections from the manuscript abstract (include quotes in quotation marks "like this" to indicate direct quotes from your manuscript), or elaborate on this item by providing additional information not in the ms, or briefly explain why the item is not applicable/relevant for your study

---

1b-iv) RESULTS section in abstract must contain use data

Report number of participants enrolled/assessed in each group, the use/uptake of the intervention (e.g., attrition/adherence metrics, use over time, number of logins etc.), in addition to primary/secondary outcomes. (Note: Only report in the abstract what the main paper is reporting. If this information is missing from the main body of text, consider adding it)

subitem not at all important 1 2 3 4 5 essential

Does your paper address subitem 1b-iv?

Copy and paste relevant sections from the manuscript abstract (include quotes in quotation marks "like this" to indicate direct quotes from your manuscript), or elaborate on this item by providing additional information not in the ms, or briefly explain why the item is not applicable/relevant for your study

---

1b-v) CONCLUSIONS/DISCUSSION in abstract for negative trials

Conclusions/Discussions in abstract for negative trials: Discuss the primary outcome - if the trial is negative (primary outcome not changed), and the intervention was not used, discuss whether negative results are attributable to lack of uptake and discuss reasons. (Note: Only report in the abstract what the main paper is reporting. If this information is missing from the main body of text, consider adding it)

subitem not at all 1 2 3 4 5 essential

important

Does your paper address subitem 1b-v?

Copy and paste relevant sections from the manuscript abstract (include quotes in quotation marks "like this" to indicate direct quotes from your manuscript), or elaborate on this item by providing additional information not in the ms, or briefly explain why the item is not applicable/relevant for your study

INTRODUCTION

2a) In INTRODUCTION: Scientific background and explanation of rationale

2a-i) Problem and the type of system/solution

Describe the problem and the type of system/solution that is object of the study: intended as stand-alone intervention vs. incorporated in broader health care program? Intended for a particular patient population? Goals of the intervention, e.g., being more cost-effective to other interventions, replace or complement other solutions? (Note: Details about the intervention are provided in “Methods” under 5)

subitem not at all important 1 2 3 4 5 essential

Does your paper address subitem 2a-i? *

Copy and paste relevant sections from the manuscript (include quotes in quotation marks "like this" to indicate direct quotes from your manuscript), or elaborate on this item by providing additional information not in the ms, or briefly explain why the item is not applicable/relevant for your study

Hemophilia
is a congenital coagulopathy characterized by a deficiency of a clotting factor (FVIII in hemophilia A; FIX in hemophilia B). Most clinical manifestations are musculoskeletal: bruising and, mainly, hemarthrosis. The recurrence of joint bleeding causes degenerative, progressive and chronic joint deterioration (hemophiliac arthropathy), chronic pain and a deteriorated perception of the patient's quality of life. Symptoms of hemophilic arthropathy include limited range of motion, decreased periarticular muscle strength, proprioception, and chronic pain [1, 2].
The ankle joint, as well as the knee and elbow joints, are the most affected part of the locomotive apparatus [3], posing important functional and proprioceptive limitations for the adult patient. These limitations are the result of developing intraarticular alterations such as joint space narrowing, the development of osteophytes, bone deformities or axial alterations [4]. Prophylactic treatment has been shown to be highly effective in the prevention of hemophilic arthropathy
[5], however many patients develop this sequela as a result of late initiation of treatment, poor adherence to prophylaxis or the development of inhibitors (antibodies to FVIII/FIX concentrates).
The approach to hemophilic ankle arthropathy includes pharmacological
and orthopedic interventions with the aim of improving the symptoms reported by the patient. The administration of anti-inflammatory and analgesic drugs [6], conducting synoviorthesis to remove the synovial membrane and, ultimately, arthrodesis of the ankle joint are the most commonly used therapeutic tools [7].
Physiotherapy can play an extremely important role in the treatment of hemophilic ankle arthropathy. Manual therapy techniques [8], therapeutic
exercise [9] or electrotherapy [10] have provided positive results in the control of chronic pain in these patients.
The degenerative process itself is one of the intrinsic causes affecting the fascial system, since it favors loss of elasticity and flexibility, and triggers a process of pathological cross-linking of collagen fibers. Fascial therapy aims to remove fascial tissue restriction through mechanical stimuli applied during 3 - 5 minutes to promote the piezoelectric effect in the crystalline matrix of the fascia [11]. The mechanical stimulus duly administered over the fascia (pressure or tangential compression) could trigger a cellular response secondary to the effect of that stimulus on the conjunctive system and promote cellular communication through mechanotransduction and piezoelectricity
mechanisms [12]. Although pilot and cohort studies [13, 14] have proved
that this technique is safe in patients with hemophilic arthropathy of the ankle, clinical studies need to be conducted with a large sample size in order to confirm that this technique is indeed suitable.
Fascia is defined as the soft tissue component of the connective tissue system that permeates the human body forming a continuous three-dimensional matrix of structural support throughout the body [15].
Myofascial release is a physiotherapy technique whereby sustained pressure is applied in order to reduce restrictions of the fascial

Hemophilia is a congenital coagulopathy characterized by a deficiency of a clotting factor (FVIII in hemophilia A; FIX in hemophilia B). Most clinical manifestations are musculoskeletal: bruising and, mainly, hemarthrosis. The recurrence of joint bleeding causes degenerative, progressive and chronic joint deterioration (hemophiliac arthropathy), chronic pain and a deteriorated perception of the patient's quality of life. Symptoms of hemophilic arthropathy include limited range of motion, decreased periarticular muscle strength, proprioception, and chronic pain [1, 2].

The ankle joint, as well as the knee and elbow joints, are the most affected part of the locomotive apparatus [3], posing important functional and proprioceptive limitations for the adult patient. These limitations are the result of developing intraarticular alterations such as joint space narrowing, the development of osteophytes, bone deformities or axial alterations [4]. Prophylactic treatment has been shown to be highly effective in the prevention of hemophilic arthropathy [5], however many patients develop this sequela as a result of late initiation of treatment, poor adherence to prophylaxis or the development of inhibitors (antibodies to FVIII/FIX concentrates).

The approach to hemophilic ankle arthropathy includes pharmacological and orthopedic interventions with the aim of improving the symptoms reported by the patient. The administration of anti-inflammatory and analgesic drugs [6], conducting synoviorthesis to remove the synovial membrane and, ultimately, arthrodesis of the ankle joint are the most commonly used therapeutic tools [7].

Physiotherapy can play an extremely important role in the treatment of hemophilic ankle arthropathy. Manual therapy techniques [8], therapeutic exercise [9] or electrotherapy [10] have provided positive results in the control of chronic pain in these patients.

The degenerative process itself is one of the intrinsic causes affecting the fascial system, since it favors loss of elasticity and flexibility, and triggers a process of pathological cross-linking of collagen fibers. Fascial therapy aims to remove fascial tissue restriction through mechanical stimuli applied during 3 - 5 minutes to promote the piezoelectric effect in the crystalline matrix of the fascia [11]. The mechanical stimulus duly administered over the fascia (pressure or tangential compression) could trigger a cellular response secondary to the effect of that stimulus on the conjunctive system and promote cellular communication through mechanotransduction and piezoelectricity mechanisms [12]. Although pilot and cohort studies [13, 14] have proved that this technique is safe in patients with hemophilic arthropathy of the ankle, clinical studies need to be conducted with a large sample size in order to confirm that this technique is indeed suitable.

Fascia is defined as the soft tissue component of the connective tissue system that permeates the human body forming a continuous three-dimensional matrix of structural support throughout the body [15].

Myofascial release is a physiotherapy technique whereby sustained pressure is applied in order to reduce restrictions of the fascial system. Mechanical stimulation favors the readjustment of collagen fibers by improving the quality of movement, fluid circulation and lymphatic drainage [16]. These changes can help improve adhesions caused by processes such as scarring and fibrosis in the body region to be treated. Myofascial induction is a manual physiotherapy technique that applies the principles of biomechanical loading of the soft tissue and modification of the neural reflexes, through stimulation of the mechanoreceptors in the fascia with the purpose of releasing fascial restrictions and restoring healthy tissue [17].

Self-Myofascial release with a foam roller is used to address fascial adhesions and to restore the normal extensibility of soft tissues [18]. Self-Myofascial release has similar effects to those provided by manual fascial release techniques. These benefits include easing muscle tension and stiffness, reducing pain, inflammation and muscle spasms, and increasing joint mobility. Myofascial release techniques have been used to treat adhesions of the soft tissue, to relieve pain and reduce tissue sensitivity, edema, and inflammation, while improving muscle recovery [19].

2a-ii) Scientific background, rationale: What is known about the (type of) system

Scientific background, rationale: What is known about the (type of) system that is the object of the study (be sure to discuss the use of similar systems for other conditions/diagnoses, if appropiate), motivation for the study, i.e. what are the reasons for and what is the context for this specific study, from which stakeholder viewpoint is the study performed, potential impact of findings [2]. Briefly justify the choice of the comparator.

subitem not at all important 1 ( ) 2 ( ) 3 (•) 4 ( ) 5 ( ) essential

Does your paper

Copy and paste relevant sections from the manuscript

address subitem 2a-ii? *

(include quotes in quotation marks "like this" to indicate direct quotes from your manuscript), or elaborate on this item by providing additional information not in the ms, or briefly explain why the item is not applicable/relevant for your study

For
Self-Myofascial release, patients use their own body weight on a foam
roller or on the lower limbs to exert pressure on the affected soft
tissues [20,21]. Scientific evidence shows that these types of tools
help improve the range of motion and pre and post exercise muscle
performance [21, 22]. The effectiveness of these types of exercises to
improve joint mobility has been tested by authors such as MacDonald et
al. [20] in relation to improving mobility in knee flexion, and by
Skarabot et al. [23] relative to the improvement of ankle joint
mobility.
The myofascial system possibly responds to the use of foam rollers based
on post-intervention changes in joint range of motion and pressure pain
thresholds. These changes may be due to a mechanical and
neurophysiological response [24, 25]. The direct pressure of the roller
may produce a local mechanical and global neurophysiological effect
relaxing the tissues and reducing pain in the target and surrounding
area [26].
Local pressure from the roller may affect the viscoelastic properties of
myofascia which may be responsible for such changes. Other mechanisms
that may be involved include thixotropy (reduced viscosity), myofascial
restriction, fluid-related changes, and cellular responses [24].
Researchers have also found that foam-rolling reduces arterial
stiffness, increases arterial tissue perfusion, and improves vascular
endothelial functions related to tissue relaxation [27].

2b) In INTRODUCTION: Specific objectives or hypotheses

Does your paper address CONSORT subitem 2b? *

Copy and paste relevant sections from the manuscript (include quotes in quotation marks "like this" to indicate direct quotes from your manuscript), or elaborate on this item by providing additional information not in the ms, or briefly explain why the item is not applicable/relevant for your study

The
object of this paper is to detail our approach to the design and implementation of this protocol for self-myofascial release using a Foam
Roller applied in patients with hemophilic ankle arthropathy looking to
reduce the frequency of joint bleeding and improve variables such as
range of motion, joint pain, functionality, joint state, and muscle
flexibility and strength.

METHODS

3a) Description of trial design (such as parallel, factorial) including allocation ratio

Does your paper address CONSORT subitem 3a? *

Copy and paste relevant sections from the manuscript (include quotes in quotation marks "like this" to indicate direct quotes from your manuscript), or elaborate on this item by providing additional information not in the ms, or briefly explain why the item is not

applicable/relevant for your study

A
randomized, multicenter clinical trial in patients with
hemophilia and
ankle hemophilic arthropathy. Figure 1 presents an overview
of the
study. The primary aim of this randomized trial
(ClinicalTrials.gov
NCT03914287) is to establish the safety and effectiveness of
a
physiotherapy intervention through self-myofascial release
using a Foam
Roller. Once eligibility is be determined, participants will be
randomized either to the intervention arm (experimental
group) or the
control group.

3b) Important changes to methods after trial commencement (such as eligibility criteria), with reasons

Does your paper address CONSORT subitem 3b? *

Copy and paste relevant sections from the manuscript (include quotes in quotation marks "like this" to indicate direct quotes from your manuscript), or elaborate on this item by providing additional information not in the ms, or briefly explain why the item is not applicable/relevant for your study

Not available

3b-i) Bug fixes, Downtimes, Content Changes

Bug fixes, Downtimes, Content Changes: ehealth systems are often dynamic systems. A description of changes to methods therefore also includes important changes made on the intervention or comparator during the trial (e.g., major bug fixes or changes in the functionality or content) (5-iii) and other “unexpected events” that may have influenced study design such as staff changes, system failures/downtimes, etc. [2].

subitem not at all important 1 2 3 4 5 essential

Does your paper address subitem 3b-i?

Copy and paste relevant sections from the manuscript (include quotes in quotation marks "like this" to indicate direct quotes from your manuscript), or elaborate on this item by providing additional information not in the ms, or briefly explain why the item is not applicable/relevant for your study

4a) Eligibility criteria for participants

Does your paper address CONSORT subitem 4a? *

Copy and paste relevant sections from the manuscript (include quotes in quotation marks "like this" to indicate direct quotes from your manuscript), or elaborate on this item by providing additional information not in the ms, or briefly explain why the item is not applicable/relevant for your study

The
 inclusion criteria to participate in the present study are
patients:
diagnosed with hemophilia A and B disease; over 18
years-old; diagnosed
with bilateral hemophilic arthropathy of the ankle (more than
three
points of joint damage on the Hemophilia Joint Health Score);
and on
prophylactic or on demand treatment with FVIII/FIX
concentrates.
Patients who will be excluded from the study are those:
without
ambulation ability; with inhibitors (antibodies to FVIII or FIX);
with
neurological or cognitive alterations that prevent
understanding the
questionnaires and physical tests; and who have failed to sign
the
informed consent document.

4a-i) Computer / Internet literacy

Computer / Internet literacy is often an implicit “de facto” eligibility criterion - this should be explicitly clarified.

subitem not at all important 1 2 3 4 5 essential

Does your paper address subitem 4a-i?

Copy and paste relevant sections from the manuscript (include quotes in quotation marks "like this" to indicate direct quotes from your manuscript), or elaborate on this item by providing additional information not in the ms, or briefly explain why the item is not applicable/relevant for your study

After
 obtaining the patients' informed consent, the baseline assessments
will
 be carried out at the relevant centers of the Hemophilia Associations
included in the study.

4a-ii) Open vs. closed, web-based vs. face-to-face assessments:

Open vs. closed, web-based vs. face-to-face assessments: Mention how participants were recruited (online vs. offline), e.g., from an open access website or from a clinic, and clarify if this was a purely web-based trial, or there were face-to-face components (as part of the intervention or for assessment), i.e., to what degree got the study team to know the participant. In online-only trials, clarify if participants were quasi-anonymous and whether having multiple identities was possible or whether technical or logistical measures (e.g., cookies, email confirmation, phone calls) were used to detect/prevent these.

subitem not at all important 1 2 3 4 5 essential

Does your paper address subitem 4a-ii? *

Copy and paste relevant sections from the manuscript (include quotes in quotation marks "like this" to indicate direct quotes from your manuscript), or elaborate on this item by providing additional information not in the ms, or briefly explain why the item is not applicable/relevant for your study

4a-iii) Information giving during recruitment

Information given during recruitment. Specify how participants were briefed for recruitment and in the informed consent procedures (e.g., publish the informed consent documentation as appendix, see also item X26), as this information may have an effect on user self-selection, user expectation and may also bias results.

subitem not at all important 1 2 3 4 5 essential

Does your paper address subitem 4a-iii?

Copy and paste relevant sections from the manuscript (include quotes in quotation marks "like this" to indicate direct quotes from your manuscript), or elaborate on this item by providing additional information not in the ms, or briefly explain why the item is not applicable/relevant for your study

4b) Settings and locations where the data were collected

Does your paper address CONSORT subitem 4b? *

Copy and paste relevant sections from the manuscript (include quotes in quotation marks "like this" to indicate direct quotes from your manuscript), or elaborate on this item by providing additional information not in the ms, or briefly explain why the item is not applicable/relevant for your study

After obtaining the patients' informed consent, the baseline assessments will be carried out at the relevant centers of the Hemophilia Associations included in the study. Each patient will perform the interventions at home.

4b-i) Report if outcomes were (self-)assessed through online questionnaires

Clearly report if outcomes were (self-)assessed through online questionnaires (as common in web-based trials) or otherwise.

subitem not at all important 1 2 3 4 5 essential

Does your paper address subitem 4b-i? *

Copy and paste relevant sections from the manuscript (include quotes in quotation marks "like this" to indicate direct quotes from your manuscript), or elaborate on this item by providing additional information not in the ms, or briefly explain why the item is not applicable/relevant for your study

Measurements will be performed by a physiotherapist blinded to patient assignment.
After the treatment period and at 3 months following completion, the patients included in both groups (experimental and control) will be reevaluated by the same rater, under the same conditions as the initial evaluation.
Seven dependent variables (range of motion, joint pain, functionality, joint state, muscle strength and flexibility, and activity record) will be measured at the various study assessment times (baseline, post-treatment and follow-up). The outcome measures used for these variables will be:
- Visual analogic scale (VAS). This scale will be used to evaluate the perception of joint pain, where ankle joint pain ranges from 0 to 10 points (no pain to maximum perceived pain).
- Pressure algometer (model Wagner FPN100, Wagner Instruments, CT, USA).

Measurements
will be performed by a physiotherapist blinded to patient assignment.
After the treatment period and at 3 months following completion, the
patients included in both groups (experimental and control) will be
reevaluated by the same rater, under the same conditions as the initial
evaluation.
Seven dependent variables (range of motion, joint pain, functionality,
joint state, muscle strength and flexibility, and activity record) will
be measured at the various study assessment times (baseline,
post-treatment and follow-up). The outcome measures used for these
variables will be:
- Visual analogic scale (VAS). This scale will be used to evaluate the
perception of joint pain, where ankle joint pain ranges from 0 to 10
points (no pain to maximum perceived pain).
- Pressure algometer (model Wagner FPN100, Wagner Instruments, CT, USA).
Measuring instrument used for the assessment of pressure pain, both at
the joint and remote sites (in other parts of the body) [28]. This
device uses Newton/cm2 to measure the pressure point at which the
subject perceives pain upon pressure. Pressure will be made on the
chosen site, gradually increasing at an approximate speed of 50kPa/s
until the patient reports that the sensation is becoming painful [29].
In patients with ankle arthropathy, bilateral measurements will be taken
ventral to the lateral malleolus (ankle) [30]; and at two remote points
distant from the ankle: on the spinous process of L5 and at the
extensor carpi radialis longus (on the forearm, 5 cm distal to the
lateral epicondyle of the humerus) [28].
- Activity register [31]. Using a portable instrument for assessing
physical activity, the patient's level of physical activity and main
related variables (average number of steps per day, average distance per
day, average amount of active time and energy consumption) shall be
recorded.
- Hemophilia Joint Health Score (HJHS) [32]. This measuring instrument
will be used to evaluate the joint condition of knees, ankles, and
elbows. It includes 8 items (inflammation and its duration, pain,
atrophy and muscle strength, crepitus, and reduced flexion and
extension) ranging from 0 to 20 points per joint (the higher the score,
the greater the joint deterioration).
- 6-Minute Walking test (6-MWT) [33]. The 6-MWT test is a
performance-based test that measures walking speed and the submaximal
capacity to perform an exercise. This instrument was developed for use
in patients with respiratory disease and heart failure but has been used
in children and adults with a variety of chronic conditions, including
hemophilia [34-36]. Patients are instructed to walk along a 30 to
50-meter track for 6 minutes attempting to cover as much distance as
possible without running. The distance traveled in 6 minutes is
recorded, allowing the use of a walking aid or orthopedic devices.
- Digital goniometer. Ankle range of motion shall be measured using a
digital goniometer according to protocol rules designed by Thornton et
al. [37]; the assessment can be made with the patient standing. The axis
of the goniometer will be placed parallel to the external malleolus of
the ankle. The fixed arm will be parallel to the fibula, while the
mobile arm parallel to the fifth metatarsal bone. This measuring
instrument allows more accurate measurements than those obtained with a
universal plastic goniometer. The patient will be asked to perform three
repetitions of each movement.
- Dynamometry. The maximum isometric strength of the plantar flexor
muscles of the ankles will be evaluated on both limbs with a manual
dynamometer. The patient will be placed in supine position with his or
her foot arranged at 90º of dorsal flexion. The dynamometer will be
located proximal to the metatarsophalangeal joints on the plantar side).
The patient will be asked to perform 3 isometric maximum 5-second
contractions, with a 30-second break between them, against the
dynamometer held by the evaluator [38].
- Fingertip-to-Floor Test [39]. This test is used to assess the degree
of flexibility of the posterior muscles of the lower limbs. The value is
calculated as the distance between the fingertip and the floor when
maximum hip flexion with knee extension is performed.

4b-ii) Report how institutional

Report how institutional affiliations are displayed to potential participants [on ehealth media], as affiliations with prestigious hospitals or universities

affiliations are displayed

may affect volunteer rates, use, and reactions with regards to an intervention.(Not a required item – describe only if this may bias results)

subitem not at all important 1 2 3 4 5 essential

Does your paper address subitem 4b-ii?

Copy and paste relevant sections from the manuscript (include quotes in quotation marks "like this" to indicate direct quotes from your manuscript), or elaborate on this item by providing additional information not in the ms, or briefly explain why the item is not applicable/relevant for your study

5) The interventions for each group with sufficient details to allow replication, including how and when they were actually administered

5-i) Mention names, credential, affiliations of the developers, sponsors, and owners

Mention names, credential, affiliations of the developers, sponsors, and owners [6] (if authors/evaluators are owners or developer of the software, this needs to be declared in a "Conflict of interest" section or mentioned elsewhere in the manuscript).

subitem not at all important 1 2 3 4 5 essential

Does your paper address subitem 5-i?

Copy and paste relevant sections from the manuscript (include quotes in quotation marks "like this" to indicate direct quotes from your manuscript), or elaborate on this item by providing additional information not in the ms, or briefly explain why the item is not applicable/relevant for your study

Each
 session will last approximately 15 minutes, with five physiotherapy
sessions over a period of 3 months. The Self-Myofascial release protocol
 for the lower limbs using a Foam Roller and Solid Ball Massage, adapted
 to patients with hemophilic ankle arthropathy, will include self-myofascial release of the plantar region, back of the leg, hamstring region, and adductor, abductor and pelvitrochanteric muscles.
Table 1 shows the Physiotherapy protocol used in patients with
hemophilic ankle arthropathy.

5-ii) Describe the history/development process

Describe the history/development process of the application and previous formative evaluations (e.g., focus groups, usability testing), as these will have an impact on adoption/use rates and help with interpreting results.

subitem not at all important 1 2 3 4 5 essential

Copy and paste relevant sections from the manuscript

Does your paper address subitem 5-ii?

(include quotes in quotation marks "like this" to indicate direct quotes from your manuscript), or elaborate on this item by providing additional information not in the ms, or briefly explain why the item is not applicable/relevant for your study

5-iii) Revisions and updating

Revisions and updating. Clearly mention the date and/or version number of the application/intervention (and comparator, if applicable) evaluated, or describe whether the intervention underwent major changes during the evaluation process, or whether the development and/or content was "frozen" during the trial. Describe dynamic components such as news feeds or changing content which may have an impact on the replicability of the intervention (for unexpected events see item 3b).

subitem not at all important 1 2 3 4 5 essential

Does your paper address subitem 5-iii?

Copy and paste relevant sections from the manuscript (include quotes in quotation marks "like this" to indicate direct quotes from your manuscript), or elaborate on this item by providing additional information not in the ms, or briefly explain why the item is not applicable/relevant for your study

Each
session will last approximately 15 minutes, with five physiotherapy
sessions over a period of 3 months. Each patient will perform the
interventions at home. The Self-Myofascial release protocol for the
lower limbs using a Foam Roller and Solid Ball Massage, adapted to
patients with hemophilic ankle arthropathy, will include self-myofascial
release of the plantar region, back of the leg, hamstring region, and
adductor, abductor and pelvitrochanteric muscles. Table 1 shows the
Physiotherapy protocol used in patients with hemophilic ankle arthropathy.

5-iv) Quality assurance methods

Provide information on quality assurance methods to ensure accuracy and quality of information provided [1], if applicable.

subitem not at all important 1 2 3 4 5 essential

Does your paper address subitem 5-iv?

Copy and paste relevant sections from the manuscript (include quotes in quotation marks "like this" to indicate direct quotes from your manuscript), or elaborate on this item by providing additional information not in the ms, or briefly explain why the item is not applicable/relevant for your study

5-v) Ensure replicability by publishing the source code, and/or providing

Ensure replicability by publishing the source code, and/or providing screenshots/screen-capture

screenshots/screen-capture video, and/or providing flowcharts of the algorithms used

video, and/or providing flowcharts of the algorithms used. Replicability (i.e., other researchers should in principle be able to replicate the study) is a hallmark of scientific reporting.

subitem not at all important 1 2 3 4 5 essential

Does your paper address subitem 5-v?

Copy and paste relevant sections from the manuscript (include quotes in quotation marks "like this" to indicate direct quotes from your manuscript), or elaborate on this item by providing additional information not in the ms, or briefly explain why the item is not applicable/relevant for your study

Table 1

5-vi) Digital preservation

Digital preservation: Provide the URL of the application, but as the intervention is likely to change or disappear over the course of the years; also make sure the intervention is archived (Internet Archive, webcitation.org, and/or publishing the source code or screenshots/videos alongside the article). As pages behind login screens cannot be archived, consider creating demo pages which are accessible without login.

subitem not at all important 1 2 3 4 5 essential

Does your paper address subitem 5-vi?

Copy and paste relevant sections from the manuscript (include quotes in quotation marks "like this" to indicate direct quotes from your manuscript), or elaborate on this item by providing additional information not in the ms, or briefly explain why the item is not applicable/relevant for your study

https://inhefis.com/proyecto-he-foam/

5-vii) Access

Access: Describe how participants accessed the application, in what setting/context, if they had to pay (or were paid) or not, whether they had to be a member of specific group. If known, describe how participants obtained “access to the platform and Internet” [1]. To ensure access for editors/reviewers/readers, consider to provide a “backdoor” login account or demo mode for reviewers/readers to explore the application (also important for archiving purposes, see vi).

subitem not at all important 1 2 3 4 5 essential

Does your paper address subitem 5-vii? *

Copy and paste relevant sections from the manuscript (include quotes in quotation marks "like this" to indicate direct quotes from your manuscript), or elaborate on this item by providing additional information not in the ms, or briefly explain why the item is not applicable/relevant for

your study

Participants
 will have free access, downloading the mobile app, without having to be
 members of any group. Access to the platform was approved after signing
 the informed consent. Once the study is complete, the contents of the
application (Table 1) will be posted on the website (www.inhefis.org.)

5-viii) Mode of delivery, features/functionalities/components of the intervention and comparator, and the theoretical framework

Describe mode of delivery, features/functionalities/components of the intervention and comparator, and the theoretical framework [6] used to design them (instructional strategy [1], behaviour change techniques, persuasive features, etc., see e.g., [7, 8] for terminology). This includes an in-depth description of the content (including where it is coming from and who developed it) [1],” whether [and how] it is tailored to individual circumstances and allows users to track their progress and receive feedback” [6]. This also includes a description of communication delivery channels and – if computer-mediated communication is a component – whether communication was synchronous or asynchronous [6]. It also includes information on presentation strategies [1], including page design principles, average amount of text on pages, presence of hyperlinks to other resources, etc. [1].

subitem not at all important 1 2 3 4 5 essential

Does your paper address subitem 5-viii? *

Copy and paste relevant sections from the manuscript (include quotes in quotation marks "like this" to indicate direct quotes from your manuscript), or elaborate on this item by providing additional information not in the ms, or briefly explain why the item is not applicable/relevant for your study

Participants
 will have free access, downloading the mobile app, without having to be
 members of any group. Access to the platform was approved after signing
 the informed consent. Once the study is complete, the contents of the
application (Table 1) will be posted on the website (www.inhefis.org.)

5-ix) Describe use parameters

Describe use parameters (e.g., intended “doses” and optimal timing for use). Clarify what instructions or recommendations were given to the user, e.g., regarding timing, frequency, heaviness of use, if any, or was the intervention used ad libitum.

subitem not at all important 1 2 3 4 5 essential

Does your paper address subitem 5-ix?

Copy and paste relevant sections from the manuscript (include quotes in quotation marks "like this" to indicate direct quotes from your manuscript), or elaborate on this item by providing additional information not in the ms, or briefly explain why the item is not applicable/relevant for your study

Participants
will have free access, downloading the mobile app, without having to be
members of any group. Access to the platform was approved after signing
the informed consent. Once the study is complete, the contents of the
application (Table 1) will be posted on the website (www.inhefis.org.)

5-x) Clarify the level of human involvement

Clarify the level of human involvement (care providers or health professionals, also technical assistance) in the e-intervention or as co-intervention (detail number and expertise of professionals involved, if any, as well as “type of assistance offered, the timing and frequency of the support, how it is initiated, and the medium by which the assistance is delivered”. It may be necessary to distinguish between the level of human involvement required for the trial, and the level of human involvement required for a routine application outside of a RCT setting (discuss under item 21 – generalizability).

subitem not at all important 1 2 3 4 5 essential

Does your paper address subitem 5-x?

Copy and paste relevant sections from the manuscript (include quotes in quotation marks "like this" to indicate direct quotes from your manuscript), or elaborate on this item by providing additional information not in the ms, or briefly explain why the item is not applicable/relevant for your study

5-xi) Report any prompts/reminders used

Report any prompts/reminders used: Clarify if there were prompts (letters, emails, phone calls, SMS) to use the application, what triggered them, frequency etc. It may be necessary to distinguish between the level of prompts/reminders required for the trial, and the level of prompts/reminders for a routine application outside of a RCT setting (discuss under item 21 – generalizability).

subitem not at all important 1 2 3 4 5 essential

Does your paper address subitem 5-xi? *

Copy and paste relevant sections from the manuscript (include quotes in quotation marks "like this" to indicate direct quotes from your manuscript), or elaborate on this item by providing additional information not in the ms, or briefly explain why the item is not applicable/relevant for

your study

A
 periodic follow-up will be made, through phone calls and SMS, to
answer
 questions from patients regarding the use of the application, etc.

5-xii) Describe any co-interventions (incl. training/support)

Describe any co-interventions (incl. training/support): Clearly state any interventions that are provided in addition to the targeted eHealth intervention, as ehealth intervention may not be designed as stand-alone intervention. This includes training sessions and support [1]. It may be necessary to distinguish between the level of training required for the trial, and the level of training for a routine application outside of a RCT setting (discuss under item 21 – generalizability.

subitem not at all important 1 2 3 4 5 essential

Does your paper address subitem 5-xii? *

Copy and paste relevant sections from the manuscript (include quotes in quotation marks "like this" to indicate direct quotes from your manuscript), or elaborate on this item by providing additional information not in the ms, or briefly explain why the item is not applicable/relevant for your study

Subjects
 included in the control group will receive no physical therapy
through
self-myofascial release and will continue with their usual routine of
physical activity and exercise, following the same pharmacological
treatment regimen with FVIII/FIX. At the end of the study period
the
intervention will be applied under the same conditions as the
experimental group, analyzing the overall sample at the end of the
study.

6a) Completely defined pre-specified primary and secondary outcome measures, including how and when they were assessed

Does your paper address CONSORT subitem 6a? *

Copy and paste relevant sections from the manuscript (include quotes in quotation marks "like this" to indicate direct quotes from your manuscript), or elaborate on this item by providing additional information not in the ms, or briefly explain why the item is not applicable/relevant for your study

6a-i) Online questionnaires: describe if they were validated for online use and apply CHERRIES items to describe how the questionnaires were designed/deployed

If outcomes were obtained through online questionnaires, describe if they were validated for online use and apply CHERRIES items to describe how the questionnaires were

designed/deployed [9].

subitem not at all important 1 2 3 4 5 essential

Does your paper address subitem 6a-i?

Copy and paste relevant sections from manuscript text

-Visual
 analogic scale (VAS). This scale will be used to evaluate the
perception of joint pain, where ankle joint pain ranges from 0 to 10
points (no pain to maximum perceived pain).
-Pressure algometer (model Wagner FPN100, Wagner Instruments, CT, USA).
This device uses Newton/cm2 to measure the pressure point at which the
subject perceives pain upon pressure. Pressure will be made on the
chosen site, gradually increasing at an approximate speed of 50kPa/s
until the patient reports that the sensation is becoming painful [29].
-Activity register [31]. Using a portable instrument for assessing
physical activity, the patient's level of physical activity and main
related variables
-Hemophilia Joint Health Score (HJHS) [32]. This measuring instrument
will be used to evaluate the joint condition of knees, ankles, and
elbows.
-6-Minute Walking test (6-MWT) [33]. The 6-MWT test is a
performance-based test that measures walking speed and the submaximal
capacity to perform an exercise. This instrument was developed for use
in patients with respiratory disease and heart failure but has been used
 in children and adults with a variety of chronic conditions, including
hemophilia [34-36].
-Digital goniometer. Ankle range of motion shall be measured using a
digital goniometer according to protocol rules designed by Thornton et
al. [37]; the assessment can be made with the patient standing.
-Dynamometry. The maximum isometric strength of the plantar flexor
muscles of the ankles will be evaluated on both limbs with a manual
dynamometer.
-Fingertip-to-Floor Test [39]. This test is used to assess the degree of
 flexibility of the posterior muscles of the lower limbs.

6a-ii) Describe whether and how "use" (including intensity of use/dosage) was defined/measured/monitored

Describe whether and how "use" (including intensity of use/dosage) was defined/measured/monitored (logins, logfile analysis, etc.). Use/adoption metrics are important process outcomes that should be reported in any ehealth trial.

subitem not at all important 1 2 3 4 5 essential

Does your paper address subitem 6a-ii?

Copy and paste relevant sections from manuscript text

-
Visual analogic scale (VAS). This scale will be used to evaluate the
perception of joint pain, where ankle joint pain ranges from 0 to 10
points (no pain to maximum perceived pain).
- Pressure algometer. Pressure will be made on the chosen site,
gradually increasing at an approximate speed of 50kPa/s until the
patient reports that the sensation is becoming painful [29]. In patients
with ankle arthropathy, bilateral measurements will be taken ventral to
the lateral malleolus (ankle) [30]; and at two remote points distant
from the ankle: on the spinous process of L5 and at the extensor carpi
radialis longus (on the forearm, 5 cm distal to the lateral epicondyle
of the humerus) [28].
register [31]. Using a portable instrument for assessing physical
activity, the patient's level of physical activity and main related
variables (average number of steps per day, average distance per day,
average amount of active time and energy consumption) shall be recorded.
- Hemophilia Joint Health Score (HJHS). It includes 8 items
(inflammation and its duration, pain, atrophy and muscle strength,
crepitus, and reduced flexion and extension) ranging from 0 to 20 points
per joint (the higher the score, the greater the joint deterioration).
- Minute Walking test (6-MWT) [33]. This instrument was developed for
use in patients with respiratory disease and heart failure but has been
used in children and adults with a variety of chronic conditions,
including hemophilia [34-36]. Patients are instructed to walk along a 30
to 50-meter track for 6 minutes attempting to cover as much distance as
possible without running. The distance traveled in 6 minutes is
recorded, allowing the use of a walking aid or orthopedic devices.
- Digital goniometer. The axis of the goniometer will be placed parallel
to the external malleolus of the ankle. The fixed arm will be parallel
to the fibula, while the mobile arm parallel to the fifth metatarsal
bone. This measuring instrument allows more accurate measurements than
those obtained with a universal plastic goniometer. The patient will be
asked to perform three repetitions of each movement.
- Dynamometry.The patient will be placed in supine position with his or
her foot arranged at 90º of dorsal flexion. The dynamometer will be
located proximal to the metatarsophalangeal joints on the plantar side).
The patient will be asked to perform 3 isometric maximum 5-second
contractions, with a 30-second break between them, against the
dynamometer held by the evaluator [38].
- Fingertip-to-Floor Test [39]. The value is calculated as the distance
between the fingertip and the floor when maximum hip flexion with knee
extension is performed.

6a-iii) Describe whether, how, and when qualitative feedback from participants was obtained

Describe whether, how, and when qualitative feedback from participants was obtained (e.g., through emails, feedback forms, interviews, focus groups).

subitem not at all important 1 ○ 2 ○ 3 ◉ 4 ○ 5 ○ essential

Does your paper address subitem 6a-iii?

Copy and paste relevant sections from manuscript text

6b) Any changes to trial outcomes after the trial commenced, with reasons

Does your paper address CONSORT subitem 6b? *

Copy and paste relevant sections from the manuscript (include quotes in quotation marks "like this" to indicate direct quotes from your manuscript), or elaborate on this item by providing additional information not in the ms, or briefly explain why the item is not applicable/relevant for your study

No

7a) How sample size was determined

NPT: When applicable, details of whether and how the clustering by care provides or centers was addressed

7a-i) Describe whether and how expected attrition was taken into account when calculating the sample size

Describe whether and how expected attrition was taken into account when calculating the sample size.

subitem not at all important 1 2 3 4 5 essential

Does your paper address subitem 7a-i?

Copy and paste relevant sections from manuscript title (include quotes in quotation marks "like this" to indicate direct quotes from your manuscript), or elaborate on this item by providing additional information not in the ms, or briefly explain why the item is not applicable/relevant for your study

The
sample size is justified with respect to the prevalence of patients
with congenital coagulopathies (2,039 patients with hemophilia A and B,
and von Willebrand disease in Spain).
Based on the selection criteria established (n = 612, as per the census
of the Spanish Federation of Hemophilia) the sample size at national
level would total 236 subjects, with a confidence level of 95% and an
expected dropout ratio of 15%.
Thus, a sample of 70 patients from the seven Spanish study regions is
established for the treatment and follow-up period (September 2020 –
June 2021).

7b) When applicable, explanation of any interim analyses and stopping guidelines

Does your paper address CONSORT subitem 7b? *

Copy and paste relevant sections from the manuscript (include quotes in quotation marks "like this" to indicate direct quotes from your manuscript), or elaborate on this item by providing additional information not in the ms, or briefly explain why the item is not applicable/relevant for your study

No

8a) Method used to generate the random allocation sequence

NPT: When applicable, how care providers were allocated to each trial group

Does your paper address CONSORT subitem 8a? *

Copy and paste relevant sections from the manuscript (include quotes in quotation marks "like this" to indicate direct quotes from your manuscript), or elaborate on this item by providing additional information not in the ms, or briefly explain why the item is not applicable/relevant for your study

Yes

8b) Type of randomisation; details of any restriction (such as blocking and block size)

Does your paper address CONSORT subitem 8b? *

Copy and paste relevant sections from the manuscript (include quotes in quotation marks "like this" to indicate direct quotes from your manuscript), or elaborate on this item by providing additional information not in the ms, or briefly explain why the item is not applicable/relevant for your study

For those patients who, fulfilling the study selection criteria, wish to voluntarily participate in the study, a random assignment using opaque envelopes will be made to each of the two study groups: experimental group and control group. Random assignment of patients will be carried out after cluster recruitment (based on hemophilia type and age) to ensure greater homogeneity of the study groups. This assignment will be carried out by a person not involved in the study objectives and always unaware of the identity of the subjects included in the study.

9) Mechanism used to implement the random allocation sequence (such as sequentially numbered containers), describing any steps taken to conceal the sequence until interventions were assigned

Does your paper address CONSORT subitem 9? *

Copy and paste relevant sections from the manuscript (include quotes in quotation marks "like this" to indicate direct quotes from your manuscript), or elaborate on this item by providing additional information not in the ms, or briefly explain why the item is not applicable/relevant for your study

For
those patients who, fulfilling the study selection criteria, wish to
voluntarily participate in the study, a random assignment using opaque
envelopes will be made to each of the two study groups: experimental
group and control group. Random assignment of patients will be carried
out after cluster recruitment (based on hemophilia type and age) to
ensure greater homogeneity of the study groups. This assignment will be
carried out by a person not involved in the study objectives and always
unaware of the identity of the subjects included in the study.

10) Who generated the random allocation sequence, who enrolled participants, and who assigned participants to interventions

Does your paper address CONSORT subitem 10? *

Copy and paste relevant sections from the manuscript (include quotes in quotation marks "like this" to indicate direct quotes from your manuscript), or elaborate on this item by providing additional information not in the ms, or briefly explain why the item is not applicable/relevant for your study

Yes

11a) If done, who was blinded after assignment to interventions (for example, participants, care providers, those assessing outcomes) and how

NPT: Whether or not administering co-interventions were blinded to group assignment

11a-i) Specify who was blinded, and who wasn’t

Specify who was blinded, and who wasn’t. Usually, in web-based trials it is not possible to blind the participants [1, 3] (this should be clearly acknowledged), but it may be possible to blind outcome assessors, those doing data analysis or those administering co-interventions (if any).

subitem not at all important 1 2 3 4 5 essential

Does your paper address subitem 11a-i? *

Copy and paste relevant sections from the manuscript (include quotes in quotation marks "like this" to indicate direct quotes from your manuscript), or elaborate on this item by providing additional information not in the ms, or briefly explain why the item is not applicable/relevant for your study

After
obtaining the patients' informed consent, the baseline assessments will
be carried out at the relevant centers of the Hemophilia Associations
included in the study. Measurements will be performed by a physiotherapist blinded to patient assignment.

11a-ii) Discuss e.g., whether participants knew which intervention was the

Informed consent procedures (4a-ii) can create biases and certain expectations - discuss

"intervention of interest" and which one was the "comparator"

e.g., whether participants knew which intervention was the "intervention of interest" and which one was the "comparator".

subitem not at all important 1 2 3 4 5 essential

Does your paper address subitem 11a-ii?

Copy and paste relevant sections from the manuscript (include quotes in quotation marks "like this" to indicate direct quotes from your manuscript), or elaborate on this item by providing additional information not in the ms, or briefly explain why the item is not applicable/relevant for your study

11b) If relevant, description of the similarity of interventions

(this item is usually not relevant for ehealth trials as it refers to similarity of a placebo or sham intervention to a active medication/intervention)

Does your paper address CONSORT subitem 11b? *

Copy and paste relevant sections from the manuscript (include quotes in quotation marks "like this" to indicate direct quotes from your manuscript), or elaborate on this item by providing additional information not in the ms, or briefly explain why the item is not applicable/relevant for your study

No

12a) Statistical methods used to compare groups for primary and secondary outcomes

NPT: When applicable, details of whether and how the clustering by care providers or centers was addressed

Does your paper address CONSORT subitem 12a? *

Copy and paste relevant sections from the manuscript (include quotes in quotation marks "like this" to indicate direct quotes from your manuscript), or elaborate on this item by providing additional information not in the ms, or briefly explain why the item is not applicable/relevant for your study

Yes

12a-i) Imputation techniques to deal with attrition / missing

Imputation techniques to deal with attrition / missing values: Not all participants will use the intervention/comparator as intended and attrition is typically high in ehealth trials. Specify how

values

participants who did not use the application or dropped out from the trial were treated in the statistical analysis (a complete case analysis is strongly discouraged, and simple imputation techniques such as LOCF may also be problematic [4]).

subitem not at all important 1 2 3 4 5 essential

Does your paper address subitem 12a-i? *

Copy and paste relevant sections from the manuscript (include quotes in quotation marks "like this" to indicate direct quotes from your manuscript), or elaborate on this item by providing additional information not in the ms, or briefly explain why the item is not applicable/relevant for your study

The
main descriptive statistics will be obtained (mean and standard
deviation) of the evaluations carried out for all the dependent variables. An ANOVA of partially repeated measures will be carried out
to compare the two groups (experimental and control) at the three
assessment times: baseline (T0), posttreatment (T1) and follow-up (T2).
When the interaction is significant, pairwise comparison tests will be
performed on the group factor and on the repeated measurements factor.
To control the significance level of error rate, the Bonferroni correction method will be applied. The results of the F test depend on
the significance of Mauchly's sphericity test. If significant, the Greenhouse-Geisser correction will be used. To assess clinical
relevance, we will calculate the standard error of measurement (SEM) and
minimal detectable change (MDC) for each dependent variable.

12b) Methods for additional analyses, such as subgroup analyses and adjusted analyses

Does your paper address CONSORT subitem 12b? *

Copy and paste relevant sections from the manuscript (include quotes in quotation marks "like this" to indicate direct quotes from your manuscript), or elaborate on this item by providing additional information not in the ms, or briefly explain why the item is not applicable/relevant for your study

We will use the intent-to-treat method to include all subjects who were randomized in the final data analysis.

X26) REB/IRB Approval and Ethical Considerations [recommended as subheading under "Methods"] (not a CONSORT item)

X26-i) Comment on ethics committee approval

subitem not at all important 1 2 3 4 5 essential

Does your paper address subitem X26-i?

Copy and paste relevant sections from the manuscript (include quotes in quotation marks "like this" to indicate direct quotes from your manuscript), or elaborate on this item by providing additional information not in the ms, or briefly explain why the item is not applicable/relevant for your study

The
 protocol and its subsequent randomized clinical study were approved
by
the Research Ethics Committee of the University of Murcia (ID:
2428/2019).

x26-ii) Outline informed consent procedures

Outline informed consent procedures e.g., if consent was obtained offline or online (how? Checkbox, etc.?), and what information was provided (see 4a-ii). See [6] for some items to be included in informed consent documents.

subitem not at all important 1 2 3 4 5 essential

Does your paper address subitem X26-ii?

Copy and paste relevant sections from the manuscript (include quotes in quotation marks "like this" to indicate direct quotes from your manuscript), or elaborate on this item by providing additional information not in the ms, or briefly explain why the item is not applicable/relevant for your study

X26-iii) Safety and security procedures

Safety and security procedures, incl. privacy considerations, and any steps taken to reduce the likelihood or detection of harm (e.g., education and training, availability of a hotline)

subitem not at all important 1 2 3 4 5 essential

Does your paper address subitem X26-iii?

Copy and paste relevant sections from the manuscript (include quotes in quotation marks "like this" to indicate direct quotes from your manuscript), or elaborate on this item by providing additional information not in the ms, or briefly explain why the item is not applicable/relevant for your study

RESULTS

13a) For each group, the numbers of participants who were randomly assigned, received intended treatment, and were analysed for the primary outcome

NPT: The number of care providers or centers performing the intervention in each group and the number of patients treated by each care provider in each center

Does your paper address CONSORT subitem 13a? *

Copy and paste relevant sections from the manuscript (include quotes in quotation marks "like this" to indicate direct quotes from your manuscript), or elaborate on this item by providing additional information not in the ms, or briefly explain why the item is not applicable/relevant for your study

No

13b) For each group, losses and exclusions after randomisation, together with reasons

Does your paper address CONSORT subitem 13b? (NOTE: Preferably, this is shown in a CONSORT flow diagram) *

Copy and paste relevant sections from the manuscript (include quotes in quotation marks "like this" to indicate direct quotes from your manuscript), or elaborate on this item by providing additional information not in the ms, or briefly explain why the item is not applicable/relevant for your study

13b-i) Attrition diagram

Strongly recommended: An attrition diagram (e.g., proportion of participants still logging in or using the intervention/comparator in each group plotted over time, similar to a survival curve) or other figures or tables demonstrating usage/dose/engagement.

subitem not at all important 1 2 3 4 5 essential

Does your paper address subitem 13b-i?

Copy and paste relevant sections from the manuscript or cite the figure number if applicable (include quotes in quotation marks "like this" to indicate direct quotes from your manuscript), or elaborate on this item by providing additional information not in the ms, or briefly explain why the item is not applicable/relevant for your study

14a) Dates defining the periods of recruitment and follow-up

Does your paper address CONSORT subitem 14a? *

Copy and paste relevant sections from the manuscript (include quotes in quotation marks "like this" to indicate direct quotes from your manuscript), or elaborate on this item by providing additional information not in the ms, or briefly explain why the item is not applicable/relevant for your study

Thus,
a sample of 70 patients from the seven Spanish study regions is established for the treatment and follow-up period (September 2020 – June 2021).

14a-i) Indicate if critical “secular events” fell into the study period

Indicate if critical “secular events” fell into the the study period, e.g., significant changes in Internet resources available or “changes in computer hardware or Internet delivery resources”

subitem not at all important 1 2 3 4 5 essential

Does your paper address subitem 14a-i?

Copy and paste relevant sections from the manuscript (include quotes in quotation marks "like this" to indicate direct quotes from your manuscript), or elaborate on this item by providing additional information not in the ms, or briefly explain why the item is not applicable/relevant for your study

14b) Why the trial ended or was stopped (early)

Does your paper address CONSORT subitem 14b? *

Copy and paste relevant sections from the manuscript (include quotes in quotation marks "like this" to indicate direct quotes from your manuscript), or elaborate on this item by providing additional information not in the ms, or briefly explain why the item is not applicable/relevant for your study

No

15) A table showing baseline demographic and clinical characteristics for each group

NPT: When applicable, a description of care providers (case volume, qualification, expertise, etc.) and centers (volume) in each group

Does your paper address CONSORT subitem 15? *

Copy and paste relevant sections from the manuscript (include quotes in quotation marks "like this" to indicate direct quotes from your manuscript), or elaborate on this item by providing additional information not in the ms, or briefly explain why the item is not applicable/relevant for your study

Table 2

15-i) Report demographics associated with digital divide

In ehealth trials it is particularly important to report demographics associated with digital divide issues, such as age, education, gender, social-

issues

economic status, computer/Internet/ehealth literacy of the participants, if known.

subitem not at all important 1 2 3 4 5 essential

Does your paper address subitem 15-i? *

Copy and paste relevant sections from the manuscript (include quotes in quotation marks "like this" to indicate direct quotes from your manuscript), or elaborate on this item by providing additional information not in the ms, or briefly explain why the item is not applicable/relevant for your study

Table 2

16) For each group, number of participants (denominator) included in each analysis and whether the analysis was by original assigned groups

16-i) Report multiple “denominators” and provide definitions

Report multiple “denominators” and provide definitions: Report N’s (and effect sizes) “across a range of study participation [and use] thresholds” [1], e.g., N exposed, N consented, N used more than x times, N used more than y weeks, N participants “used” the intervention/comparator at specific pre-defined time points of interest (in absolute and relative numbers per group). Always clearly define “use” of the intervention.

subitem not at all important 1 2 3 4 5 essential

Does your paper address subitem 16-i? *

Copy and paste relevant sections from the manuscript (include quotes in quotation marks "like this" to indicate direct quotes from your manuscript), or elaborate on this item by providing additional information not in the ms, or briefly explain why the item is not applicable/relevant for your study

Table 3

16-ii) Primary analysis should be intent-to-treat

Primary analysis should be intent-to-treat, secondary analyses could include comparing only “users”, with the appropriate caveats that this is no longer a randomized sample (see 18-i).

subitem not at all important 1 2 3 4 5 essential

Does your paper address subitem 16-ii?

Copy and paste relevant sections from the manuscript (include quotes in quotation marks "like this" to indicate direct quotes from your manuscript), or elaborate on this item by providing additional information not in the ms, or briefly explain why the item is not applicable/relevant for your study

Yes

17a) For each primary and secondary outcome, results for each group, and the estimated effect size and its precision (such as 95% confidence interval)

Does your paper address CONSORT subitem 17a? *

Copy and paste relevant sections from the manuscript (include quotes in quotation marks "like this" to indicate direct quotes from your manuscript), or elaborate on this item by providing additional information not in the ms, or briefly explain why the item is not applicable/relevant for your study

Table 3

17a-i) Presentation of process outcomes such as metrics of use and intensity of use

In addition to primary/secondary (clinical) outcomes, the presentation of process outcomes such as metrics of use and intensity of use (dose, exposure) and their operational definitions is critical. This does not only refer to metrics of attrition (13-b) (often a binary variable), but also to more continuous exposure metrics such as "average session length". These must be accompanied by a technical description how a metric like a "session" is defined (e.g., timeout after idle time) [1] (report under item 6a).

subitem not at all important 1 2 3 4 5 essential

Does your paper address subitem 17a-i?

Copy and paste relevant sections from the manuscript (include quotes in quotation marks "like this" to indicate direct quotes from your manuscript), or elaborate on this item by providing additional information not in the ms, or briefly explain why the item is not applicable/relevant for your study

17b) For binary outcomes, presentation of both absolute and relative effect sizes is recommended

Does your paper address CONSORT subitem 17b? *

Copy and paste relevant sections from the manuscript (include quotes in quotation marks "like this" to indicate direct quotes from your manuscript), or elaborate on this item by providing additional information not in the ms, or briefly explain why the item is not applicable/relevant for your study

No

18) Results of any other analyses performed, including subgroup analyses and adjusted analyses, distinguishing pre-specified from exploratory

Does your paper address CONSORT subitem 18? *

Copy and paste relevant sections from the manuscript (include quotes in quotation marks "like this" to indicate direct quotes from your manuscript), or elaborate on this item by providing additional information not in the ms, or briefly explain why the item is not applicable/relevant for your study

Tables 4 and 5

18-i) Subgroup analysis of comparing only users

A subgroup analysis of comparing only users is not uncommon in ehealth trials, but if done, it must be stressed that this is a self-selected sample and no longer an unbiased sample from a randomized trial (see 16-iii).

subitem not at all important 1 2 3 4 5 essential

Does your paper address subitem 18-i?

Copy and paste relevant sections from the manuscript (include quotes in quotation marks "like this" to indicate direct quotes from your manuscript), or elaborate on this item by providing additional information not in the ms, or briefly explain why the item is not applicable/relevant for your study

19) All important harms or unintended effects in each group

(for specific guidance see CONSORT for harms)

Does your paper address CONSORT subitem 19? *

Copy and paste relevant sections from the manuscript (include quotes in quotation marks "like this" to indicate direct quotes from your manuscript), or elaborate on this item by providing additional information not in the ms, or briefly explain why the item is not applicable/relevant for your study

For
those patients who, fulfilling the study selection criteria, wish to
voluntarily participate in the study, a random assignment using opaque
envelopes will be made to each of the two study groups: experimental
group and control group. Random assignment of patients will be carried
out after cluster recruitment (based on hemophilia type and age) to
ensure greater homogeneity of the study groups. This assignment will be
carried out by a person not involved in the study objectives and always
unaware of the identity of the subjects included in the study.

19-i) Include privacy breaches, technical problems

Include privacy breaches, technical problems. This does not only include physical "harm" to participants, but also incidents such as perceived or real privacy breaches [1], technical problems, and other unexpected/unintended incidents. "Unintended effects" also includes unintended positive effects [2].

subitem not at all important 1 2 3 4 5 essential

Does your paper address subitem 19-i?

Copy and paste relevant sections from the manuscript (include quotes in quotation marks "like this" to indicate direct quotes from your manuscript), or elaborate on this item by providing additional information not in the ms, or briefly explain why the item is not applicable/relevant for your study

19-ii) Include qualitative feedback from participants or observations from staff/researchers

Include qualitative feedback from participants or observations from staff/researchers, if available, on strengths and shortcomings of the application, especially if they point to unintended/unexpected effects or uses. This includes (if available) reasons for why people did or did not use the application as intended by the developers.

subitem not at all important 1 2 3 4 5 essential

Does your paper address subitem 19-ii?

Copy and paste relevant sections from the manuscript (include quotes in quotation marks "like this" to indicate direct quotes from your manuscript), or elaborate on this item by providing additional information not in the ms, or briefly explain why the item is not applicable/relevant for your study

DISCUSSION

22) Interpretation consistent with results, balancing benefits and harms, and considering other relevant evidence

NPT: In addition, take into account the choice of the comparator, lack of or partial blinding, and unequal expertise of care providers or centers in each group

22-i) Restate study questions and summarize the answers suggested by the

Restate study questions and summarize the answers suggested by the data, starting

data, starting with primary outcomes and process outcomes (use)

with primary outcomes and process outcomes (use).

subitem not at all important 1 2 3 4 5 essential

Does your paper address subitem 22-i? *

Copy and paste relevant sections from the manuscript (include quotes in quotation marks "like this" to indicate direct quotes from your manuscript), or elaborate on this item by providing additional information not in the ms, or briefly explain why the item is not applicable/relevant for your study

The
goal of the study is to evaluate the safety and effectiveness of a
protocol related to self-myofascial release with a Foam Roller when used
in patients with hemophilic ankle arthropathy.
This project is the most ambitious physiotherapy study in terms of
methodology recorded to date. The recruitment of 70 patients with
hemophilia from 5 different regions for participation in a Physiotherapy
program confers this project an extraordinary statistical power in a
rare pathology such as hemophilia. Patients with hemophilic ankle
arthropathy will receive treatment based on self-myofascial release
using a Foam Roller. Coordination among different regions of Spain, with
the participation of Universities, Hospitals and Associations of
patients, is an uncommon effort in the field of hemophilia-related
Physiotherapy research. The research team is made up of
multidisciplinary hemophilia specialists. Renowned physiotherapists with
extensive clinical experience and researchers in the field of
hemophilia, as well as experts in methodology and statistics, make up a
highly competitive and efficient multidisciplinary team.
Another potential strength of this protocol is to demonstrate the safety
and efficacy of self-myofascial release with a Foam Roller for the
treatment of hemophilia ankle arthropathy. This protocol can help to
establish a rapid, safe and effective intervention for these patients.
In addition to the clinical improvement, it would favor a greater
adherence to physiotherapeutic treatment and a better perception of
quality of life.
Self-myofascial release using a Foam Roller requires no economic
investment in cases of manual therapy. Validating its safety and
efficacy could promote the development of a quick, inexpensive and
simple physiotherapy intervention, easily achieving a widespread use.

22-ii) Highlight unanswered new questions, suggest future research

Highlight unanswered new questions, suggest future research.

subitem not at all important 1 2 3 4 5 essential

Does your paper address subitem 22-ii?

Copy and paste relevant sections from the manuscript (include quotes in quotation marks "like this" to indicate direct quotes from your manuscript), or elaborate on this item by providing additional information not in the ms, or briefly explain why the item is not applicable/relevant for your study

The
goal of the study is to evaluate the safety and effectiveness of a
protocol related to self-myofascial release with a Foam Roller when used
in patients with hemophilic ankle arthropathy.
This project is the most ambitious physiotherapy study in terms of
methodology recorded to date. The recruitment of 70 patients with
hemophilia from 5 different regions for participation in a Physiotherapy
program confers this project an extraordinary statistical power in a
rare pathology such as hemophilia. Patients with hemophilic ankle
arthropathy will receive treatment based on self-myofascial release
using a Foam Roller. Coordination among different regions of Spain, with
the participation of Universities, Hospitals and Associations of
patients, is an uncommon effort in the field of hemophilia-related
Physiotherapy research. The research team is made up of
multidisciplinary hemophilia specialists. Renowned physiotherapists with
extensive clinical experience and researchers in the field of
hemophilia, as well as experts in methodology and statistics, make up a
highly competitive and efficient multidisciplinary team.
Another potential strength of this protocol is to demonstrate the safety
and efficacy of self-myofascial release with a Foam Roller for the
treatment of hemophilia ankle arthropathy. This protocol can help to
establish a rapid, safe and effective intervention for these patients.
In addition to the clinical improvement, it would favor a greater
adherence to physiotherapeutic treatment and a better perception of
quality of life.
Self-myofascial release using a Foam Roller requires no economic
investment in cases of manual therapy. Validating its safety and
efficacy could promote the development of a quick, inexpensive and
simple physiotherapy intervention, easily achieving a widespread use.

20) Trial limitations, addressing sources of potential bias, imprecision, and, if relevant, multiplicity of analyses

20-i) Typical limitations in ehealth trials

Typical limitations in ehealth trials: Participants in ehealth trials are rarely blinded. Ehealth trials often look at a multiplicity of outcomes, increasing risk for a Type I error. Discuss biases due to non-use of the intervention/usability issues, biases through informed consent procedures, unexpected events.

subitem not at all important 1 2 3 4 5 essential

Does your paper address subitem 20-i? *

Copy and paste relevant sections from the manuscript (include quotes in quotation marks "like this" to indicate direct quotes from your manuscript), or elaborate on this item by providing additional information not in the ms, or briefly explain why the item is not applicable/relevant for your study

Not yet

21) Generalisability (external validity, applicability) of the trial findings

NPT: External validity of the trial findings according to the intervention, comparators,

patients, and care providers or centers involved in the trial

21-i) Generalizability to other populations

Generalizability to other populations: In particular, discuss generalizability to a general Internet population, outside of a RCT setting, and general patient population, including applicability of the study results for other organizations

subitem not at all important 1 2 3 4 5 essential

Does your paper address subitem 21-i?

Copy and paste relevant sections from the manuscript (include quotes in quotation marks "like this" to indicate direct quotes from your manuscript), or elaborate on this item by providing additional information not in the ms, or briefly explain why the item is not applicable/relevant for your study

21-ii) Discuss if there were elements in the RCT that would be different in a routine application setting

Discuss if there were elements in the RCT that would be different in a routine application setting (e.g., prompts/reminders, more human involvement, training sessions or other co-interventions) and what impact the omission of these elements could have on use, adoption, or outcomes if the intervention is applied outside of a RCT setting.

subitem not at all important 1 2 3 4 5 essential

Does your paper address subitem 21-ii?

Copy and paste relevant sections from the manuscript (include quotes in quotation marks "like this" to indicate direct quotes from your manuscript), or elaborate on this item by providing additional information not in the ms, or briefly explain why the item is not applicable/relevant for your study

OTHER INFORMATION

23) Registration number and name of trial registry

Does your paper address CONSORT subitem 23? *

Copy and paste relevant sections from the manuscript (include quotes in quotation marks "like this" to indicate direct quotes from your manuscript), or elaborate on this item by providing additional information not in the ms, or briefly explain why the item is not applicable/relevant for your study

Registration: Clinicaltrials.gov NCT00102401

24) Where the full trial protocol can be accessed, if available

Does your paper address CONSORT subitem 24? *

Cite a Multimedia Appendix, other reference, or copy and paste relevant sections from the manuscript (include quotes in quotation marks "like this" to indicate direct quotes from your manuscript), or elaborate on this item by providing additional information not in the ms, or briefly explain why the item is not applicable/relevant for your study

Table 1

25) Sources of funding and other support (such as supply of drugs), role of funders

Does your paper address CONSORT subitem 25? *

Copy and paste relevant sections from the manuscript (include quotes in quotation marks "like this" to indicate direct quotes from your manuscript), or elaborate on this item by providing additional information not in the ms, or briefly explain why the item is not applicable/relevant for your study

The
study has been approved by the Ethics Committee of the
University of
Murcia (ID: 2428/2019). Patient recruitment will begin in
September
2020, with an intervention period until June 2021. Data collection
will
take place between September 2020 and October 2021, recruiting
70
patients with hemophilia.

X27) Conflicts of Interest (not a CONSORT item)

X27-i) State the relation of the study team towards the system being evaluated

In addition to the usual declaration of interests (financial or otherwise), also state the relation of the study team towards the system being evaluated, i.e., state if the authors/evaluators are distinct from or identical with the developers/sponsors of the intervention.

subitem not at all important 1 2 3 4 5 essential

Does your paper address subitem X27-i?

Copy and paste relevant sections from the manuscript (include quotes in quotation marks "like this" to indicate direct quotes from your manuscript), or elaborate on this item by providing additional information not in the ms, or briefly explain why the item is not applicable/relevant for your study

About the CONSORT EHEALTH checklist

As a result of using this checklist, did you make changes in your manuscript? *

- yes, major changes
- yes, minor changes
- no

What were the most important changes you made as a result of using this checklist?

How much time did you spend on going through the checklist INCLUDING making changes in your manuscript *

20

As a result of using this checklist, do you think your manuscript has improved? *

- yes
- no
- Otro:

Would you like to become involved in the CONSORT EHEALTH group?

This would involve for example becoming involved in participating in a workshop and writing an "Explanation and Elaboration" document

- yes
- no

Otro:

Any other comments or questions on CONSORT EHEALTH

STOP - Save this form as PDF before you click submit

To generate a record that you filled in this form, we recommend to generate a PDF of this page (on a Mac, simply select "print" and then select "print as PDF") before you submit it.

When you submit your (revised) paper to JMIR, please upload the PDF as supplementary file.

Don't worry if some text in the textboxes is cut off, as we still have the complete information in our database. Thank you!

Final step: Click submit !

Click submit so we have your answers in our database!

Enviar

Nunca envíes contraseñas a través de Formularios de Google.

Este contenido no ha sido creado ni aprobado por Google. Notificar uso inadecuado - Términos del Servicio - Política de Privacidad

Formularios
